# Performance of a new instrument designed to assess medication use in cognitive impairment and dementia: A cross‐sectional analysis

**DOI:** 10.1002/alz.087439

**Published:** 2025-01-09

**Authors:** Rishabh Sharma, Linda Lee, Feng Chang, Tejal Patel

**Affiliations:** ^1^ University of Waterloo, Waterloo, ON Canada; ^2^ Schlegel‐UW Research Institute on Aging, Waterloo, ON Canada; ^3^ MINT Memory Clinic, Kitchener, ON Canada

## Abstract

**Background:**

The Medication Review in Cognitive Impairment and Dementia (MedRevCiD) checklist is a new tool designed to assist health care professionals in optimizing medication use in individuals with Mild Cognitive Impairment (MCI) or dementia. It consists of 6 domains, each of which addresses a specific medication use issue such as medication management and adherence. The primary objective of this study was to compare the mean number of drug‐related problems (DRPs) identified with MedRevCiD Checklist to the Medication Appropriateness Index (MAI) in older adults attending a primary care‐based memory clinic.

**Methods:**

A cross‐sectional analysis was conducted at a Multi‐speciality Interprofessional Team‐based (MINT) memory clinic. A medication review was conducted by applying the MAI initially, followed by application of the MedRevCiD checklist to assess medication use and to identify DRPs for participants enrolled in the study. The Wilcoxon signed‐rank test was used to determine if a significant difference exists in the average number of DRPs per person as identified through MedRevCiD compared to MAI.

**Results:**

A total of 44 participants with a mean age of 80.2 ± 6.2 years enrolled in the study. Of the participants, 45.5% (20/44) were female, 36.4% (16/44) had mild cognitive impairment, 20.5% (9/44) had mixed dementia and 11.4% (5/44) had vascular dementia. Participants had an average of 6.7 ± 3.4 comorbidities, most commonly hypertension (63.6%, 28/44), hyperlipidemia (31.8%, 14/44), chronic kidney disease (27.2%, 12/44) and obstructive sleep apnea (25%, 11/44). Participants were taking a median of 7.5 medications (interquartile range 6) per person. A total of 134 DRPs were identified with the use of the MedRevCiD as compared with 81 with the use of the MAI (mean 3.05 ± 4.0 with MedRevCiD vs 1.84 ± 2.9 with MAI; p<0.001). Over 50% of the DRPs identified with the MedRevCiD fell within Domain 6 which focuses on optimizing medication use, while the majority of the DRPs identified from the MAI were focused on drug/disease interactions.

**Conclusion:**

Findings provide insight into the frequency of DRPs among older adults with MCI or dementia. The MedRevCiD checklist proved to be a valuable tool with heightened ability in detecting DRPs in this population.

